# Asomatognosia: Structured Interview and Assessment of Visuomotor Imagery

**DOI:** 10.3389/fpsyg.2020.544544

**Published:** 2021-01-14

**Authors:** Gianluca Saetta, Olivia Zindel-Geisseler, Franziska Stauffacher, Carlo Serra, Gilles Vannuscorps, Peter Brugger

**Affiliations:** ^1^Neuropsychology Unit, University Hospital Zurich, Zurich, Switzerland; ^2^Department of Psychology, University of Zurich, Zurich, Switzerland; ^3^Psychiatric University Clinic Zurich, Zurich, Switzerland; ^4^Neuropsychology Unit, Rehabilitation Centre Valens, Valens, Switzerland; ^5^Department of Neurosurgery, University Hospital Zurich, Zurich, Switzerland; ^6^Institute of Psychological Sciences and Institute of Neuroscience, Université catholique de Louvain, Louvain-la-Neuve, Belgium

**Keywords:** asomatognosia, bodily self-consciousness, neuropsychological assessment, visuomotor imagery, right parietal lobe

## Abstract

Asomatognosia designates the experience that one’s body has faded from awareness. It is typically a somaesthetic experience but may target the visual modality (“asomatoscopy”). Frequently associated symptoms are the loss of ownership or agency over a limb. Here, we elaborate on the rigorous nosographic classification of asomatognosia and introduce a structured interview to capture both its core symptoms and associated signs of bodily estrangement. We additionally report the case of a pure left-sided hemiasomatognosia occurring after surgical removal of a meningioma in the right atrium. Despite the wide lesions of the right angular gyrus and of the temporo-parietal junction, the patient did not present visuospatial deficits or bodily awareness disorders other than hemiasomatognosia. The patient and 10 matched controls’ motor imagery was formally assessed with a limb laterality task in which they had to decide whether hands and feet presented under different angles of rotation depicted a left or a right limb. Bayesian statistics showed that patient’s reaction times were significantly impaired exclusively for the left foot and especially for mental rotations requiring somatomotor rather than visual limb representations. This was in accordance with a more enduring left-sided hemiasomatognosia for the lower limbs confined to the somesthetic modality. Our findings shed new light on motor imagery in asomatognosia and encourage the future use of the structured interview introduced here. In addition, the limb laterality task may capture phenomenological elements of a case by chronometric means. This allows a more standardized reporting of phenomenological detail and improves communication across different clinical facilities.

## Introduction

Asomatognosia is defined as the impression that one’s own body has ceased to exist (Critchley, 1953). Most often, only one half of the body (usually the left) is affected (“hemiasomatognosia”); hence, “the characteristic feature is a subjective sensation as if there existed nothing to the left of the midline of the body” (Critchley, 1953, p. 237). This sensation is most typically a bodily feeling, i.e., the loss is somesthetic (“pure asomatognosia”), but it may, either in addition or in isolation, involve the visual modality. Thus, a patient of Carp’s felt the right half of her body absent but could convince herself that this somesthetic impression was, in fact, illusory by looking at the missing side and seeing it (somesthetic, but no visual asomatognosia; Carp, 1952). Conversely, a patient with a right thalamic tumor felt his sensation of an absent left hemibody confirmed by looking at the void body space and not seeing his left side (somesthetic *and* visual asomatognosia; Stockert, 1934). Cases in which the own body or parts of it have faded from vision but can still be felt are also described but should explicitly be referred to as *visual* asomatognosia or asomatoscopy (Magri and Mocchetti, 1967; Arzy et al., 2006).

Historically, the notion of asomatognosia as a “feeling of ‘nothingness”’ (Critchley, 1953, p. 237) has long remained undisputed. Apart from its scholarly treatment in Critchley’s seminal volume on the parietal lobes, the phenomenon was discussed at length in the French and German literature and defined in accordance with the English language definition as “sentiment d’absence d’une partie du corps” (Hécaen and de Ajuriaguerra, 1952, p. 170) or as the (illusory) experience of amputation (“sentiment d’ amputation,” Cambier et al., 1984); “Amputationserlebnis” (Menninger-Lerchenthal, 1935, p. 97–100). Mikorey (1952, passim) borrowed a term from zoology to emphasize possible evolutionary–biological roots of asomatognosia (“psychologische Autotomie,” psychological autotomy). Conceptually, in the tradition of European neurology, asomatognosia was, thus, uniformly viewed as a disorder of body schema, more specifically as a transient disruption of such a postural model of the body as proposed by Head and Holmes (1911) and previously described as “aschematia” by Bonnier (1905). In their historical review of the phenomenon, Vallar and Papagno (2003) (see also Blanke et al., 2008; Dieguez and Annoni, 2013) made it clear that pure asomatognosia is often seen in the company of related disorders of a central representation of the body or, frequently, its left side. Already Critchley (1953, p. 225) had listed such accompanying symptoms, especially different forms of unilateral neglect, anosognosia, anosodiaphoria, confabulatory denial of hemiparesis (somatoparaphrenia) and forms of sensations of “deadness” of parts of the body.

In their recent, authoritative definition paper on asomatognosia, Jenkinson et al. (2018) appreciate the felt absence as the defining feature of the phenomenon but also note the frequent association of asomatognosia with symptoms of nonrecognition or misrecognition of own body parts. This association may have confused some authors in the past as they mixed up associated symptoms and asomatognosia as originally defined. Feinberg (1997), for instance, introduced the “syndrome of asomatognosia” as “denial of ownership of the arm” (p. 130), thereby mistaking asomatognosia for somatoparaphrenia. Such blurring of related yet distinct clinical manifestations of cerebral damage can induce confusion, especially when claims to neuroanatomical correlates are made. Thus, the purported distinction between neural contributions to asomatognosia on the one hand and somatoparaphrenia on the other (Feinberg et al., 2010) turns out to be noninformative and even misleading once asomatognosia is newly defined as “unawareness of ownership of one’s arm” (p. 276). In fact, as pointed out by peer review, such violations of the original definitions may be passed on to the follow-up literature. As a consequence, somatoparaphrenia and asomatognosia have frequently been conflated after Feinberg’s (1997) influential contribution (see, e.g., Coltheart, 2007; de Vignemont, 2017).

It is against this background that we here propose a structured interview for the assessment of asomatognosia and the description of associated symptoms (Appendix). We also describe a patient with pure hemiasomatognosia after extirpation of an intraventricular meningioma in the right atrium. We provide a detailed description of the characteristics of the experience. We also report the results of a visuomotor imagery task (mental rotation of hands and feet) administered to the patient. We discuss the observed reaction time (RT) pattern with reference to the clinical symptomatology.

## Methods

### Case Report

**FIGURE 1:**
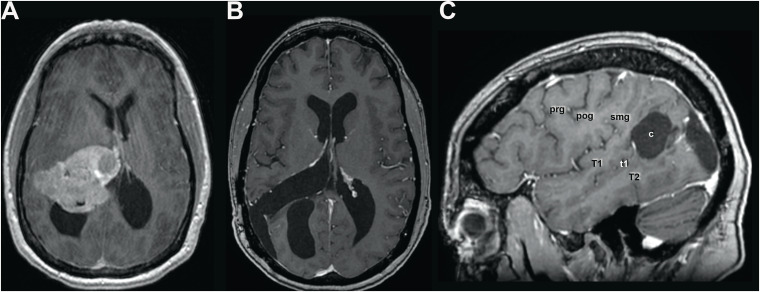
**(A)** Pre- and **(B)** postoperative axial and sagittal **(C)** MR scan with gadolinium showing the preoperative extent of the meningioma, filling the atrium and displacing anteriorly the thalamus and medially the third ventricle and velum interpositum, thereby inducing a subocclusive hydrocephalus. Given the considerable lateral extent of the lesion, a lateral transcortical transangular approach was chosen rather than an interhemispheric transprecuneal approach, which would be usually indicated for lesions in the atrium. The lesion was completely removed as illustrated in panel **(B)**. Panel **(C)** shows the location and extent of the corticectomy in the angular gyrus. c, corticectomy; pog, postcentral gyrus; prg, precentral gyrus; smg, supramarginal gyrus; T1, superior temporal gyrus; t1, superior temporal sulcus; T2, middle temporal gyrus. Scans are shown according to radiological convention (right hemisphere on left side of image).

Patient ASG (acronymic initials for *A*-*S*omato-*G*nosia) is a 53-year-old, right-handed woman with a master’s degree in business management. She is divorced and has two adult children. After an uneventful neurological and psychiatric history, somatic complaints such as headaches, vertigo, and gait disturbances as well as cognitive symptoms, such as forgetfulness and inability to concentrate, led to the discovery of an intraventricular meningioma in the right atrium (Figure 1). Its dimension and considerable lateral extension made surgical removal by an interhemispheric route appear unfavorable, and the meningioma was microsurgically removed via a transangular gyrus trajectory after right-sided temporo-parietal craniotomy.

Two or three days after surgery, ASG noticed a sensory deficit, i.e., “a slight numbness” in the left hand and foot, which alternated with the feeling of spasticity in these body parts. These sensations were waxing and waning, occurred approximately three times a day and typically lasted for some minutes. A neurological examination showed a slight spastic increase in tone in the left upper extremity, a left-sided hemi-hypoesthesia, a left-sided inferior quadrantanopia, and a benign positional vertigo. Postural sense was normal in all extremities. No neuropsychological assessment was undertaken at that time.

Four to 5 months after surgery, the patient suddenly noticed a “most peculiar feeling in the awareness of (her) body.” She experienced it for the first time when walking her dog. The left half of her body was seemingly nonexistent, “not just feeling numb or unresponsive to touch, but no longer present at all.” This feeling of absence concerned both lower and upper limbs and the left part of the head. It could be present for the leg alone but never exclusively for the arm or the head. It was typically experienced when walking, could last for a few minutes up to an hour, and was accompanied by vertigo and gait difficulties (no fall ever occurred). No sign of visual neglect seemed present, as ASG kept seeing the left side of her body, which puzzled her: “How can it be that I see something, whose absence I so convincingly feel?” ASG never experienced any such episode while standing in front of a mirror. She has never seen other people’s bodies as incomplete. The paroxysmal feeling of an absent left half-body evoked some horror when first experienced, but the patient soon got used to it, and the phenomenon lost its emotional impact. With a kind of amusement, she noticed that she was still able to lead her dog with the leash held by her left, “absent” hand. These episodes occurred several times a day (slightly more frequently in the evening compared with the morning hours) for at least 4 months. They then got spontaneously less frequent and receded by the feeling, over several weeks, of only the lower left body parts being felt as transiently absent. ASG spontaneously reported these experiences to her neurosurgeon; before, she had never talked to anybody else about them.

A neurological examination 5 months after surgery, i.e., when ASG was experiencing these episodes on a daily basis, produced results not different from the initial exploration briefly after the operation. A routine neuropsychological examination revealed a general slowing in cognitive processing speed, which manifested itself in speed-sensitive attentional and executive tasks. Moreover, a mild-to-moderate deficit in verbal and nonverbal episodic memory was evident. The only signs of parietal dysfunction were slight problems in calculation and a mild left-sided inattention in isolated tasks on spatial exploration. Performances in tasks on language, visual perception, and construction, praxis, mental rotation, and executive functions were flawless. The used neuropsychological tests and the scores obtained by the patient are reported in Table 1.

**TABLE 1:** Neuropsychological performance of U.B. (with impaired performance highlighted in bold).

Neuropsychological function	Neuropsychological test	Score (*z*-score)
**Attentional functions**		
Alertness	TAP^1^, alertness	
– Median tonic alertness (ms)		**360 (−1.7)**
– Median phasic alertness (ms)		**287 (−1.2)**
Selective attention	TAP^1^, go/nogo	
– Median (ms)		575 (−0.3)
– Error		1 (−0.3)
– Omission error		**1 (−1.7)**
Divided attention	TAP^1^, divided attention	
– Median auditiv (ms)		**800 (−2.4)**
– Median visual (ms)		**1234 (−2.5)**
– Error		2 (−0.7)
– Omission error		**12 (−2.5)**
Information processing speed		
Visuo-verbal (s)	D-KEFS^2^	
Color naming		**43 (−2.0)**
Reading		**37 (−2.7)**
– Psychomotor (s)	TMT-A^3^	**80 (<−3.2)**
**Learning and memory**		
Memory span	WMS-R^4^	
– Verbal		7 (−0.5)
– Visual		**6 (−1.5)**
Verbal-episodic memory	HVLT-R^5^	
– Learning (words/max.)		**15/36 (<−3.2)**
– Late-delay free recall (words/max.)		**6 (−2.3)**
– Recall (%)		85 (−0.8)
– Recognition		**7 (−2.7)**
Nonverbal-episodic memory	BVMT-R^6^	
– Learning (points/max.)		**17/36 (−1.2)**
– Late-delay free recall (points/max.)		**6 (−1.4)**
– Recall (%)		**60 (−3.0)**
– Recognition		**5 (−1.1)**
**Executive functions**		
Interference control	D-KEFS^2^	
– Speed (s)		**95 (−2.3)**
– Speed relative (scaled value)		**−**1 (−0.8)
– Error		1 (0.2)
Cognitive flexibility		
Visuo-verbal	D-KEFS^2^	
Speed (s)		**105 (−1.7)**
Speed relative (scaled value)		2 (0.7)
Error		1 (0.2)
Psychomotor	TMT-B^3^	
Speed (s)		**126 (<−3.2)**
Speed relative to TMT-A		1.47 (1.51)
Error		0
Fluency		
– Verbal phonematic (correct s-words)	RWT^7^	**11 (−1.7)**
– Nonverbal (correct)	H5PT^8^	19 (**−**1.0)
Working memory	WMS-R^4^	
– Verbal		8 (0.5)
– Visual		**4 (−1.8)**
**Visuo-spatial functions**		
Elementary functions of visual perception and cognition	Screening after Schnider^9^	Normal processing of orientation, figure-ground segregation, form and color. Discrete difficulties in the recognition of objects presented in non-canonical views. Normal processing of facial information, including affect.
Visuoconstruction	RCFT^10^	
– Accuracy (points/max.)		**25/36 (−2.5)**
– Speed (s)		**460 (−2.5)**
**Questionnaires**		
Depression	ADS^11^	28 (−1.0)
Fatigue	WEIMuS^12^	
– Physical		**23 (−2.8)**
– Cognitive		**27 (−3.2)**
– Total		**50 (−3)**

*^1^Test of Attentional Performance (Zimmermann and Fimm, 2007).*

*^2^Delis – Kaplan Executive Function System (Delis et al., 2001).*

*^3^Trail-Making-Test, Tombaugh (2004).*

*^4^Wechsler Memory Scale – Revised (Härting et al., 2000).*

*^5^Hopkins Verbal Learning Test – Revised (Brandt and Benedict, 2001).*

*^6^Brief Visuospatial Memory Test – Revised (Benedict, 1997).*

*^7^Regensburger verbal fluency test (Aschenbrenner et al., 2000).*

*^8^HAMASCH 5-point-test (Haid et al., 2002).*

*^9^Screening of visual functions (Schnider, 2004).*

*^10^Rey Complex Figure Test (Meyers and Meyers, 1995).*

*^11^General Depression Scale (Hautzinger and Bailer, 1992).*

*^12^Würzburger Fatigue Inventory (Flachenecker et al., 2008).*

### Assessment of Visuomotor Imagery

**FIGURE 2:**
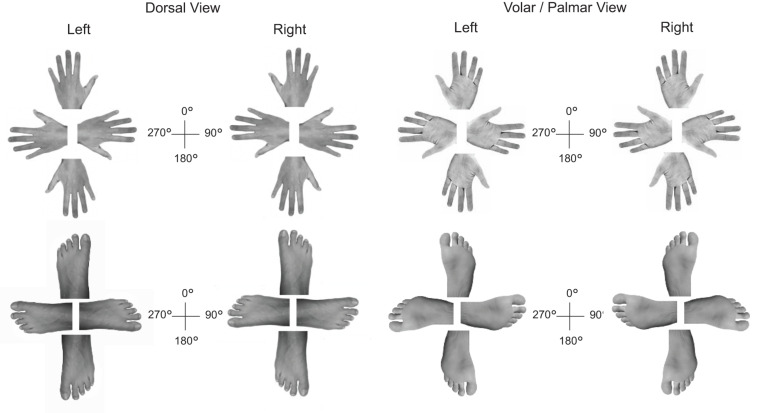
Overview of the hand (top) and foot stimuli (bottom) presented during the limb laterality task.

Approximately 2 months after the neuropsychological exam, we assessed ASG’s capacity of visuomotor imagery for body parts in a computerized mental rotation task with hands and feet as visual stimuli (Parsons, 1987). These were depicted under four angles of rotation (0°, 90°, 180°, 270°; see Figure 2). Hands and feet were shown together within the same task once in volar, once in dorsal view. All stimuli (128 in total) were presented centrally on a laptop screen and spanned a visual angle of approximately 7° to 10° horizontally. ASG and controls were required to press a left-sided (right-sided) response key with her left (right) index finger on seeing a left-sided (right-sided) limb. Stimuli were displayed until a response was given. Accuracy and speed were equally stressed. Ten practice trials, which were not analyzed, preceded the task. Feedback about response accuracy was provided exclusively during these practice trials. During the training phase, feedback on the correctness of the response appeared at the center of the screen. Stimulus presentation and response collection was programmed with the software E-prime 3.0 (Psychology Software tools).

Ten age-matched controls (six men, four women, mean age: 48.5, SD = 14.87) were tested with an identical procedure. ASG’s performance accuracy was high (mean accuracy = 92.2%) and comparable to that of the controls (group mean accuracy = 90.47%, SD = 11.25%). Only RTs of correct decisions were analyzed. We compared ASG’s RTs to those of the control group with the Bayesian inferential methods for use in single-case studies described in Crawford and Garthwaite (2007). This method proved robust for comparing one patient to a small control group. Data preprocessing and statistical analysis were performed in R studio v. 1.1.442. The Crawford – Garthwaite Bayesian test was performed with the function “Crawford.test” included in the R psycho v0.4.91 package (Makowski, 2018). The confidence interval bound was set at 95%. Significant threshold was set at 0.1 and the number of performed iterations was 10,000. Data, stimuli, E-prime program, and R scripts used for data analysis and visualization, and data can be viewed, reused, and downloaded under this link: osf.io/qmkd3/.

The study was approved by the Ethical Committee of the University of Zurich. After being informed about the purposes of the study, participants provided their written informed consent. The study was performed according to the Helsinki Declaration (1964). Data, E-prime program, stimuli, and R Scripts used for data analysis and visualization can be viewed, reused, and downloaded under this link: osf.io/qmkd3/.

## Results

**FIGURE 3:**
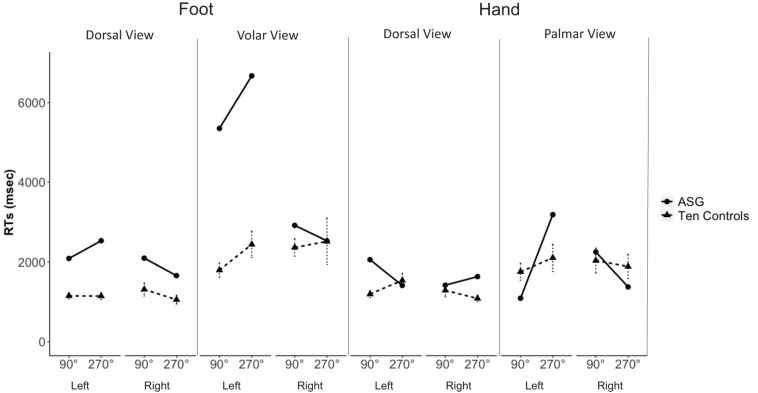
ASG’ mean RTs (dots and thick lines) and 10 control participants’ mean RTs (triangles and dashed lines; error bars indicate standard error of the mean) of correct decisions to left and right hands and feet, shown in dorsal and volar/palmar view at 90° and 270° of rotation.

While being tested, ASG did not experience hemiasomatognosia. Nevertheless, analysis of her RTs to hands and feet under the different angles of rotation revealed that ASG was slower than control participants identifying specifically *left* feet displayed in volar view for the “comfortable” 90° postures {Crawford – Garthwaite Bayesian test, controls mean RT = 1797, SD = 532, ASG’s mean RT = 5350, *z* = 6.68, percentile = 100.00, *p* < 0.001. ASG’s RTs were higher than 99.99% [95% CI (99.99, 100.00)] of the controls’ RTs}. RTs to left feet displayed in volar view were also significantly slower in ASG for the “awkward” 270° postures {Crawford – Garthwaite Bayesian test, control sample mean RT = 2440, SD = 996, ASG’s mean RT = 6670, *z* = 4.25, percentile = 100.00, *p* < 0.01. ASG’s RTs were higher than 99.83% [95% CI (99.24, 100.00)] of the controls’ RTs; see Figure 3}.

**FIGURE 4:**
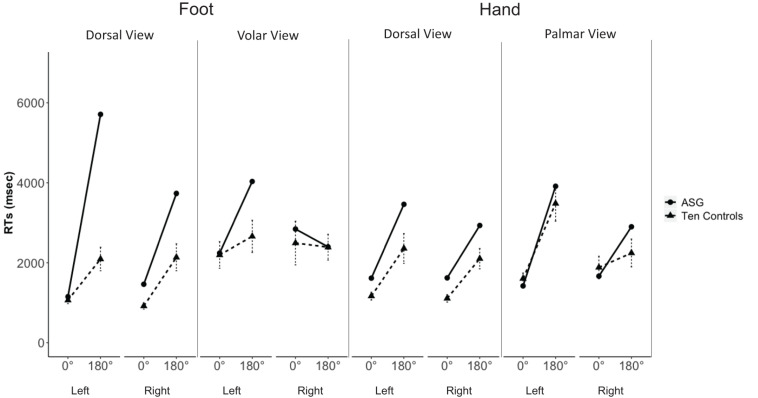
ASG’s mean RTs (dots and thick lines) and 10 control participants’ mean RTs (triangles and dashed lines; error bars indicate standard error of the mean) of correct decisions to left and right hands and feet, shown in dorsal and volar/palmar view at 0° and 180° of rotation.

Furthermore, there was a significant “inversion effect” (longer RTs to stimuli under 180° than under 0°) for feet (Figure 4); crucially, for the difficult 180° stimuli, her RTs to *left* feet shown in dorsal views were significantly longer than those of control subjects (Crawford – Garthwaite Bayesian test, control sample mean RT = 2092, SD = 932, ASG’s mean RT = 5710, *z* = 3.88, percentile = 99.99, *p* < 0.01. ASG’s RTs were higher than 99.76% [95% CI (98.84, 100.00)] of the controls’ RTs}. None of the comparisons for right limb stimuli were significant (*p* > 0.05).

## Discussion

The structured interview introduced here (see Appendix) was used for the assessment of hemiasomatognosia in patient ASG. She initially reported her paroxysmal experiences of left-sided hemiasomatognosia to the neurosurgeon. They were associated with vertigo but never led to a fall (Bonnier, 1905). The feeling of nonexistence did not encompass a change in the feelings of ownership or of agency over the affected limbs. It was confined to the somesthetic domain as ASG could still see her left body half (in contrast to cases with both somesthetic and visual asomatognosia (Stockert, 1934) or pure visual asomatognosia or asomatoscopy (Arzy et al., 2006). She also saw other people’s bodies normally; that is, she did not project own symptoms onto others (Schilder, 1919). Importantly, ASG was *not* deluded regarding her deficit in awareness of left-sided limbs. In contrast to somatoparaphrenia, asomatognosia does not involve any delusional elaboration of the misperceived bodily state (see Vallar and Ronchi, 2009).

ASG was administered a limb laterality judgment task thought to rely on the integrity of the participant’s body schema. Body schema is the implicit and continuously updated representation of the position occupied by the limbs in the space and the biomechanical constraints arising from the sensory inputs, mainly proprioceptive, and that access the motor system directly for the performance of routine motor acts (Head and Holmes, 1911). According to Parsons’ comparative method (Parsons, 1987), this task requires the activation of two processes. First, the selection of the representation of either the right or left limb and then the mental rotation maneuver of this representation to match the position of the depicted hand. Two outcome measures are typically considered for this task. The first is the accuracy, which hints of the integrity of the performer’s body schema. Here, we observed that ASG’s accuracy did not statistically differ from that of the controls, suggesting an intact body schema. The second outcome is the RTs. RT analysis may offer important insights into the disruption of one or the other process. For instance, previous studies show that experimentally induced pain or expectation of pain to a hand leads to an increase in the RT that is specific for the non-painful limb. The interpretation of this effect is that an attention bias toward the painful limb, i.e., a marked difficulty in allocating the attentional resources away from it induces a delay in the access to the non-painful limb representation (Hudson et al., 2006). On the other hand, longer RTs specific for the painful hand were observed in patients with complex regional pain syndrome (CRPS; Moseley, 2004). CRPS is characterized by a profound cortical reorganization and neglect-like sensory and exploration deficits reflected in the phenomenal experience that a “limb feels foreign” (Galer and Jensen, 1999; Förderreuther et al., 2004). Compatible with the evidence that CRPS patients take longer to recognize the limb that is felt as foreign (Moseley, 2004), ASG’s RTs to left, but not right, feet were slower than those of controls’, specifically when presented under a motorically challenging degree of rotation (180°). The similarity between CRPS and asomatognosia for the affected limb’s phenomenal experience and the time needed to identify its laterality suggest that a cortical remapping may underlie the latter disorder as much as it does for the former (Galer and Jensen, 1999; Förderreuther et al., 2004). Although this remains a speculation, future studies combining the behavioral task presented here with functional neuroimaging will bring clarity on this open question.

Because ASG presented with mild left-sided inattention on tasks of spatial exploration, we cannot exclude a causative role of attentional asymmetries in response to images of left and right limbs. Future studies should test patients with asomatognosia without neglect to specifically address this issue. Decisions to feet depicted in biomechanically awkward postures (270°) were slower than controls’ for volar views but not for dorsal views. Previous studies show that hands presented in palm view require the manipulation of the respective motor representation, and hands in back view are more likely to be visually processed. Although, admittedly, this view-dependent effect has not been explicitly tested for the mental rotation of feet, ASG’s RT pattern for feet may reflect the type of left-sided hemiasomatognosia (no visual component), and we would predict a different pattern for patients with hemiasomatoscopy (visual hemiasomatognosia). However, whether view-dependent effects for feet analogous to those described for hands do in fact exist needs to be established empirically in healthy research participants. In the only previous report of an asomatognosic patient’s mental rotation of hands (Arzy et al., 2006), composite hand – arm pictures with either compatible or incompatible laterality were used as stimuli. The authors’ patient was slower than controls in responding to the body part stimuli, whereas her mental rotation speed was comparable for letter stimuli. Patient ASG did not experience hemiasomatognosia at the time of solving the limb laterality task, yet compared with controls, her RTs were longer to some of the left-sided but to none of the right-sided limbs. That the RT differences were more obvious for foot than hand stimuli is in accordance with the observation that her asomatognosia had always been more pronounced for the leg compared to the arm and that it was still experienced for the lower limb when it no longer occurred for the upper. Also, the fact that ASG’s RTs to left-sided body parts were significantly longer than controls’ RTs under 180° rotation but not under 0° rotation speaks against the assumption that there was an unspecific slowing for left-sided body parts. Rather, this difference seems to corroborate ASG’s deficit in motor imagery. Our findings, thus, invite the use of limb laterality tasks to objectify an individual patient’s symptoms of asomatognosia.

Our study comes with an important limitation. That is, ASG’s motor function for left upper and lower limbs were only clinically tested and judged normal. A formal assessment of the motor function is lacking. Alterations in the motor function of upper and lower limbs might have an impact on the visuomotor task. However, although the responses for left-sided stimuli were given with the left hand, ASG’s RTs were significantly slower for the stimuli requiring the mental rotation of a different effector, the *left feet*, making it unlikely that impaired efferent processes might underly her impaired performance in the task.

It remains a challenge for future clinical studies to test patients with hemiasomatognosia repeatedly: once while feeling their body absent and once with a recovered, normal bodily awareness. Paradigms to experimentally induce asomatognosia in healthy volunteers may also prove revealing in clinical populations (Newport and Gilpin, 2011; Stone et al., 2018).

## Data Availability Statement

The data, stimuli, E-prime program, and R scripts used for data analysis and visualization, and data can be viewed, reused, and downloaded under this link: osf.io/qmkd3/.

## Ethics Statement

The studies involving human participants were reviewed and approved by the Ethics Committee of the University Hospital Zurich. The patients/participants provided their written informed consent to participate in this study. Written, informed consent was obtained from the individuals for the publication of any potentially identifiable images or data included in this article.

## Author Contributions

PB, GS, and OZ-G conceived the project and wrote the manuscript. GS programmed the computerized task, collected data, and performed data analysis. OZ-G and FS performed the neuropsychological assessment. CS performed surgery, and localized and described the lesion. GV significantly enriched the discussion of the findings. All the authors reviewed and approved the final version of the manuscript.

## Conflict of Interest

The authors declare that the research was conducted in the absence of any commercial or financial relationships that could be construed as a potential conflict of interest.

## Appendix

### Structured Interview

Questions should be adapted to the individual case as a patient’s initial description may be relatively precise (e.g., “it was as if I had lost any awareness of my left arm”) or exceedingly vague (e.g., “I had some very peculiar experience concerning my body”). Adaptation is also required with respect to the clinical picture within the patient’s asomatognosia reported (e.g., whether there is an associated hemiparesis/hemiplegia, hemianopia, etc.). Once it has become clear to the examiner that the patient describes an episode of asomatognosia, phenomenal detail should be inquired, if possible, by inviting free report while minimizing suggestive cues. The time course of the disturbances (and, if applicable, of single episodes) should be inquired, as (hemi)asomatognosia is commonly a long-term phenomenon (Sierra et al., 2002), and experiences in the second range may suggest an epileptic origin (So and Schäuble, 2004). Questions unrelated to the core phenomenon of “bodily nonexistence” may uncover related perturbations of somatognosis and inform about the relative involvement of the somatosensory and visual modalities, respectively. The issue of perspective transformation is crucial in cases of asomatognosia associated with transitivism (Wernicke, 1906; Schilder, 1919), i.e., the projection of own symptoms onto other persons. Will a patient who lacks awareness of her left side see other persons’ left or right side absent? The answer depends not only on lesion location but is revealing about the relationships between body, self, and phenomenal space (Brugger, 2002).

We recommend videotaping the entire interview after having obtained the patient’s written informed consent. A sketchy portrait of the patient’s own body, which depicts affected body regions may also be helpful, preferably on a back and a front view sketch of a human body. Needless to say, the interview should be complemented by a comprehensive neuropsychological evaluation. In cases of unilateral asomatognosia, testing different forms of hemispatial neglect is especially important.

### Structured Interview Template

Ask any patient, who reports an absence or disappearance of the body or a feeling of amputation the following questions (to be adapted to the individual case):

(a) Where on the body
  Which part(s) of the body appeared absent/amputated? Was the sensation of absence stronger/qualitatively different for some of these parts? Were the same body parts always affected? Can you provide a sketch of your body (front and back view) and mark the affected parts? Please use different colors/shadings for phenomenally different impressions. (If patient is reluctant to draw a self-portrait, provide two simple sketches of a human body, once in back view and once in front view).
(b) Triggering and modulating factors; modalities involved
  On first occurrence, was there anything that may have triggered the experience?
  Can the feeling of an absent body (part) be induced/modified by any factors?
  Specifically, is the feeling of absence modulated by
  – Moving the affected body part(s)?
  – Having the affected body part(s) moved by somebody else?
  – Touching the affected body part(s) with one of your unaffected limbs (if possible)?
  – Having your affected body part(s) touched by somebody else?
  – Having your eyes open/shut?
  – Looking at the affected body part(s)?
  – Any other factor?
  When you look at the body part you feel to be absent, do you still see it?
  Have you ever experienced the absence of your body (parts)…
  – When in front of a mirror? (if yes, describe what happened to your mirror image, if anything)
  – When looking at other people’s bodies? (if yes, describe any change you might have visually perceived)
  Can you deliberately induce/abolish the experience?
(c) Frequency and time course
  When was the first time you experienced an absence of your body? How many times have you had this experience since then? What is its daily (weekly/monthly) frequency? Is there a circadian pattern? How long does an episode usually last (provide minimal and maximal duration)? Do you still experience these episodes these days? Has their frequency been changing over the weeks (months/years)? Has anything else been changing?
(d) Associated symptoms
  Was the feeling of nonexistence of your body (parts) accompanied by any other sensation or feeling? (regularly? occasionally?)
  Has your consciousness been altered while experiencing the feeling of absence of your body (parts)? (expanded, dream-like, dizzy?)
  Have you ever felt the subjectively absent body parts
  – To belong to somebody else?
  – To move/behave in ways that seemed not under your control?
  – To be dead or rotting away?
  – To be detached from the body and possibly present elsewhere?
